# Evaluation of sulfobutylether-β-cyclodextrin (SBECD) accumulation and voriconazole pharmacokinetics in critically ill patients undergoing continuous renal replacement therapy

**DOI:** 10.1186/s13054-015-0753-8

**Published:** 2015-02-03

**Authors:** Tyree H Kiser, Douglas N Fish, Christina L Aquilante, Joseph E Rower, Michael F Wempe, Robert MacLaren, Isaac Teitelbaum

**Affiliations:** Department of Clinical Pharmacy, University of Colorado Skaggs School of Pharmacy and Pharmaceutical Sciences, 12850 E Montview Blvd, Mail Stop C238, Aurora, CO 80045 USA; Department of Pharmaceutical Sciences, University of Colorado Skaggs School of Pharmacy and Pharmaceutical Sciences, 12850 E Montview Blvd, Mail Stop C238, Aurora, CO 80045 USA; Department of Medicine, University of Colorado Anschutz Medical Campus, 12605 E 16th Ave, Box F774, Aurora, CO 80045 USA

## Abstract

**Introduction:**

Intravenous (IV) voriconazole is not recommended in patients with creatinine clearance <50 ml/min to avoid potentially toxic accumulation of sulfobutylether-β-cyclodextrin (SBECD). The purpose of this study was to evaluate the pharmacokinetics of SBECD, voriconazole, and voriconazole N-oxide in critically ill patients undergoing continuous renal replacement therapy (CRRT) and to determine if CRRT removes SBECD sufficiently to allow for the use of IV voriconazole without significant risk of SBECD accumulation.

**Methods:**

This prospective, open-label pharmacokinetic study enrolled patients >18 years old receiving IV voriconazole for a known or suspected invasive fungal infection while undergoing CRRT. Serial blood and effluent samples were collected on days 1, 3, 5, 7, and every 3 to 5 days thereafter. SBECD, voriconazole, and voriconazole N-oxide plasma and effluent concentrations were measured by liquid chromatography-tandem mass spectrometry. Pharmacokinetic, pharmacodynamic, and pharmacogenetic analyses were conducted.

**Results:**

Ten patients (mean ± standard deviation (SD)) 53 ± 11 years old, 50% male, 81 ± 14 kg, with Acute Physiologic and Chronic Health Evaluation II (APACHE II) scores of 31.5 ± 3.8 were evaluated. All patients underwent continuous venovenous hemofiltration (CVVH) with a median predilution replacement fluid rate of 36 (interquartile range (IQR) 32 to 37) ml/kg/hr and total ultrafiltration rate of 38 (IQR 34 to 39) ml/kg/hr. Mean ± SD voriconazole and SBECD dosages administered were 8.1 ± 2.1 mg/kg/day and 129 ± 33 mg/kg/day, respectively. Voriconazole plasma trough concentrations were >1 mg/L in all patients with CVVH accounting for only 15% of the total body clearance. CVVH accounted for 86% of the total body clearance of SBECD with the majority of the dose being recovered in the effluent. Minimal increases in dose normalized SBECD area under the concentration-time curve from 0 to 12 hours (AUC0-12) (4,484 ± 4,368 to 4,553 ± 2,880 mg*hr/L; *P* = 0.97) were observed after study day 1.

**Conclusions:**

CVVH effectively removed SBECD at a rate similar to the ultrafiltration rate. Voriconazole clearance by CVVH was not clinically significant. Standard dosages of IV voriconazole can be utilized in patients undergoing CVVH without significant risk of SBECD accumulation.

**Trial registration:**

ClinicalTrials.gov NCT01101386. Registered 6 April 2010.

**Electronic supplementary material:**

The online version of this article (doi:10.1186/s13054-015-0753-8) contains supplementary material, which is available to authorized users.

## Introduction

Voriconazole is a triazole antifungal that has a broad spectrum of activity against yeast, molds, and dimorphic fungi. Voriconazole is currently recommended as the first-line therapy for patients with invasive *Aspergillus* infection and can also be utilized to treat patients with other life-threatening systemic mycoses [1,2]. Since voriconazole has limited water solubility, the intravenous (IV) voriconazole formulation includes the vehicle sulfobutylether-beta-cyclodextrin sodium (SBECD). SBECD is comprised of a multitude of polymeric structures with varying degrees of substitution. SBECD has a molecular mass of approximately 2,163 Da, is not protein bound, has a volume of distribution (Vd) similar to extracellular water (0.2 L/kg), and is predominately excreted by glomerular filtration in the kidney [3,4]. In patients with normal renal function, SBECD is effectively eliminated with a half-life of less than 2 hours, but accumulation of SBECD is known to occur in patients with a creatinine clearance (CrCl) <50 ml/min [5]. Accumulation of SBECD in animals at doses 50-fold greater (3,000 mg/kg) than typically administered in humans has been associated with liver necrosis and obstruction of the renal tubules [3]. These toxicities have not yet been observed in humans; however, it is recommended that oral voriconazole be utilized instead of IV voriconazole in patients with a CrCl <50 ml/min [5]. Therefore, clinicians must either face the potential risk of SBECD accumulation or choose an alternative antifungal therapy in these patients.

Patients with severe systemic fungal infections are commonly treated in the intensive care unit (ICU). These patients frequently have complicated comorbidities including bone marrow or solid organ transplant, acute kidney injury, multiorgan dysfunction, and shock. Unfortunately, absorption of enterally administered antimicrobials can be erratic, leading to inadequate plasma concentrations and treatment failure in some patients [6-14]. Many ICU patients also have relative contraindications to taking oral medications, including gastrointestinal bleeding, mucositis, inadequate oral access, nonfunctioning gastrointestinal (GI) tract, or gastroparesis. Therefore, many clinicians prefer to administer voriconazole intravenously in these patients.

In addition, many critically ill patients with acute kidney injury are hypotensive and cannot tolerate the blood flow rates or fluid shifts that occur with intermittent dialysis therapy. Therefore, critically ill patients commonly receive continuous renal replacement therapy (CRRT). This mode is highly effective at removing fluid and solute from blood. It is known that SBECD is removed by intermittent hemodialysis at a rate similar to a CrCl of 55 ml/min, and that accumulation of SBECD occurs only during the time periods when dialysis is not being provided to the patient [4,15]. Thus, it is plausible that CRRT would be effective at removing SBECD, thereby allowing for the safe administration of IV voriconazole to these critically ill patients. Therefore, the primary objective of this study was to determine if CRRT can adequately remove the SBECD vehicle from the plasma so that IV voriconazole may be utilized in critically ill patients with renal dysfunction. Secondarily, the pharmacokinetics of IV voriconazole and its N-oxide metabolite, influences of cytochrome P450 (CYP) 2C19 phenotype on voriconazole and its N-oxide metabolite pharmacokinetics, and adverse effects of SBECD accumulation were also evaluated.

## Materials and methods

### Patients and ethics

This study was an open-label, single-center, descriptive pharmacokinetic evaluation at the University of Colorado Hospital. The decision to administer IV voriconazole was made by the attending physician and was not dictated by the study. From May 2010 to December 2012, patients >18 years of age who were receiving CRRT and were prescribed IV voriconazole therapy for the treatment of a fungal infection were considered eligible for study participation. Patients expected to be on CRRT or intravenous voriconazole therapy for less than 5 days were excluded.

This study was approved by the Colorado Multiple Institutional Review Board. Informed consent and health insurance portability and accountability act authorization was obtained from the patient’s designated proxy prior to enrollment.

### Continuous renal replacement therapy

All patients underwent CRRT utilizing the NxStage™ System One dialysis machine (NxStage Medical Inc. Lawrence, MA, USA) with NxStage Cartridge Express and filter (high-flux polyethersulfone membrane with 1.5 m^2^ membrane surface area). Continuous venovenous hemofiltration (CVVH) was the mode utilized in all patients. Blood flow rates ranged from 200 to 300 ml/min and predilution replacement therapy fluid flow rates were between 2,000 and 6,000 ml/hr. Dialysis initiation, settings, and net ultrafiltration were prescribed by the consulting nephrology service. CRRT was performed without the use of citrate anticoagulation.

### Voriconazole administration and sample collection

Patients were started on voriconazole per routine standard of care at a loading dose of 6 mg/kg IV every 12 hours x 2 doses, then 4 mg/kg IV every 12 hours thereafter. Voriconazole maintenance doses could be reduced by 50% in patients with Child Pugh A or B hepatic disease per package insert recommendations.

On days 1, 3, and 5 patients underwent pharmacokinetic sampling for determination of SBECD, voriconazole, and voriconazole N-oxide concentrations. Plasma and effluent samples were collected at 0, 0.5, 1, 2, 3, 4, 6, 8, and 12 hours. If the patient remained on CVVH at day 7, plasma and dialysate samples were collected at time 0, 2, and 8 hours on that day. Thereafter, sparse sampling occurred every 3 to 5 days. Blood samples were immediately centrifuged at 3,000 g and plasma and effluent samples were stored at −80°C until analysis.

### Quantification of SBECD, voriconazole, and voriconazole N-oxide concentrations

SBECD, voriconazole, and voriconazole N-oxide concentrations were determined utilizing a liquid chromatography-tandem mass spectrometry (LC-MS/MS) method at the University of Colorado Anschutz Medical Campus Medicinal Chemistry Core Facility (detailed methodology can be found in Additional file 1).

### Pharmacokinetic analysis

Plasma and dialysate concentration-time data for SBECD, voriconazole, and voriconazole N-oxide were analyzed by standard noncompartmental pharmacokinetics (pharmacokinetic analysis methodology described in detail in Additional file 1). All calculations were made by programming pharmacokinetic equations into Microsoft Excel 2010 (Microsoft Corporation, Redmond, WA, USA) and were validated using WinNonlin version 5.0.1 (Pharsight Corporation, Mountain View, CA, USA). To evaluate drug accumulation, dose-normalized area under the concentration-time curve (AUC), maximum drug concentration in the plasma (Cmax), and minimum drug concentration in the plasma (Cmin) were evaluated on study day 1 versus study days ≥3 and compared with a paired *t* test.

### Pharmacokinetic modeling

The pharmacokinetic parameters total systemic clearance (CL_s_) and Vd were calculated for SBECD using population pharmacokinetic techniques in ADAPT V software (Biomedical Simulation Resource, Los Angeles, CA, USA). Pharmacokinetic parameters were calculated for the entire population and for the various levels of CVVH ultrafiltration (2,000, 3,000, and 6,000 ml/hr), using a one-compartment, short infusion model. The population pharmacokinetic parameters and associated error were then utilized to simulate expected concentrations for the various levels of CVVH in ADAPT V, using the individual simulation with output error option selected. The dose used for simulation represented a typical administration of SBECD (96 mg/kg IV every 12 hours for two doses, followed by 64 mg/kg IV every 12 hours) for an 80 kg individual, with a one-hour infusion length. Concentrations were simulated every hour, starting at first dose and continuing through steady state. Data from these simulations were then plotted using GraphPad Prism Software (version 5.04, La Jolla, CA, USA).

### Monte Carlo simulations for probability of target attainment

Monte Carlo simulation (Crystal Ball version 7, Oracle Corporation, Redwood Shores, CA, USA) was used to calculate probability of target attainment (PTA) for pharmacodynamic goals. The model randomly applied values for CL_s_, Vd, weight, and unbound (42% unbound (range 36 to 48%)) voriconazole AUC0 to 12 (*f*AUC0-12) derived from data obtained from the study patients. Minimum inhibitory concentration (MIC) distributions obtained from the Clinical and Laboratory Standards Institute (CLSI), the European Committee on Antimicrobial Susceptibility Testing (EUCAST), or provided by Pfizer pharmaceuticals were placed into our Monte Carlo simulation for comparison to voriconazole plasma concentrations and pharmacokinetic variability (isolate numbers and MIC distributions by database can be found in Table S1 in Additional file 1). Five thousand simulations were performed at each MIC value and for each of the selected pathogens. The probability of target attainment for *Candida albicans*, *C. glabrata*, *C. krusei*, *C. parapsilosis*, *C. tropicalis*, *Aspergillus fumigatus*, *A. niger*, *and A. terreus* were evaluated for a goal *f*AUC/MIC >25 or >35 at a voriconazole loading dose of 6 mg/kg IV every 12 hours and maintenance dosages of 4 and 6 mg/kg every 12 hours. If >90% target obtainment was not observed, continued analyses of loading and maintenance doses of 8 mg/kg, 10 mg/kg, and 12 mg/kg every 12 hours were conducted.

### Cytochrome P450 (CYP) 2C19 pharmacogenetic analysis

Genetic polymorphisms in *CYP2C19*, the principal enzyme that metabolizes voriconazole, were interrogated in all study patients. Specifically, CYP2C19*2 (loss-of-function), CYP2C19*3 (loss-of-function), and CYP2C19*17 (gain-of-function) polymorphisms were genotyped using PCR-pyrosequencing (pharmacogenetic for consistency analysis described in more detail within Additional file 1). CYP2C19 metabolizing enzyme phenotypes (that is, ultrarapid, extensive, intermediate, or poor metabolizers) were assigned based on genotypes using literature conventions [16].

### Safety end point and adverse effect monitoring

Patients were assessed for potential adverse effects associated with SBECD accumulation, with a focus on renal and hepatic function tests as these toxicities have been demonstrated in animal studies. Serum creatinine (SCr) values were monitored and worsening or reversal of renal dysfunction was assessed in each patient during and after discontinuation of CVVH. Renal function recovery was evaluated by assessment of SCr values and the need for continued intermittent hemodialysis therapy after ICU and hospital discharge. To assess hepatic function, alterations in aspartate aminotransferase, alanine aminotransferase, total bilirubin, albumin, total protein, and international normalized ratio were evaluated in each patient.

## Results

Ten patients were enrolled. Patients were (mean ± standard deviation (SD)) 53 ± 11 years old, 50% male, 81 ± 14 kg, and had Acute Physiologic and Chronic Health Evaluation II score (APACHE II) scores of 31.5 ± 3.8. Patient demographics are reported in Table 1 and patient classification based upon *CYP2C19* genotypes and phenotypes are presented in Table 2. All enrolled patients were critically ill with multiple comorbidities and were receiving IV voriconazole for the treatment of a known or suspected systemic fungal infection. All patients received CVVH with a median predilution replacement fluid rate of 36 (IQR 32 to 37) ml/kg/hr and ultrafiltration rate of 38 (IQR 34 to 39) ml/kg/hr. Patients had minimal residual renal function and remained anuric or oliguric during the entire study period. Mean ± SD voriconazole and SBECD dosages administered were 8.1 ± 2.1 mg/kg/day and 129 ± 33 mg/kg/day. Patients were enrolled in the study for a mean of 5 ± 3 days (range 2 to 11 days).

**Table 1:** **Patient characteristics**

	**Age (years)**	**Weight (kg)**	**Sex**	**Race**	**APACHE II**	**SOFA**	**Voriconazole indication**
Mean ± SD	53 ± 11	81 ± 14	50% Male	70% White	32 ± 4	16 ± 4	Pulmonary aspergillosis (n = 4)
				10% Asian			Empiric broad spectrum antifungal (n = 4)
Median (range)	55 (33–70)	83 (54–100)		10% Hispanic/White	31 (27–38)	17 (9–20)	Scedosporium brain abscess (n = 1)
				10% Native American			*Candida glabrata* peritonitis (n = 1)

Data are presented as mean ± standard deviation (SD); median (minimum to maximum range); or numbers/proportions. APACHE II, Acute Physiologic and Chronic Health Evaluation II score; SOFA, Sequential Organ Failure Assessment score.

**Table 2:** **Patient genotype and phenotype**

**Patient**	***CYP2C19*** **genotype**	**CYP2C19 phenotype assignment**
1	*1/*1	Extensive metabolizer
2	*1/*17	Ultrarapid metabolizer
3	*1/*1	Extensive metabolizer
4	*1/*1	Extensive metabolizer
5	*1/*2	Intermediate metabolizer
6	*1/*1	Extensive metabolizer
7	*1/*1	Extensive metabolizer
8	*2/*17	Intermediate metabolizer
9	*1/*1	Extensive metabolizer
10	*1/*2	Intermediate metabolizer

An asterisk (*) followed by a numeral represents each allele in the given genotype. *CYP2C19*, cytochrome P450 2C19 enzyme.

**Figure 1:**
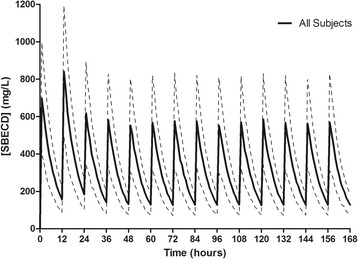
**Simulated plasma SBECD pharmacokinetic profiles in all subjects.** Data represent 1,000 patient simulations for SBECD exposure with voriconazole 6 mg/kg IV every 12 hours for two doses followed by 4 mg/kg IV every 12 hours from time 0 to day 7. Data presented as mean concentration (solid line) and standard deviation (dashed lines). SBECD, sulfobutylether-β-cyclodextrin; IV, intravenous.

**Figure 2:**
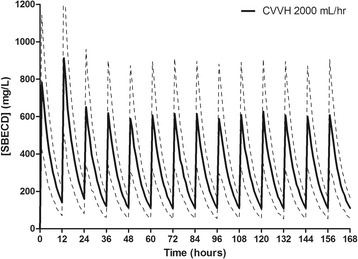
**Simulated plasma SBECD pharmacokinetic profiles in subjects undergoing CVVH with an ultrafiltration rate of 2,000 ml/hr.** Data represent 1,000 patient simulations for SBECD exposure with voriconazole 6 mg/kg IV every 12 hours for two doses followed by 4 mg/kg IV every 12 hours from time 0 to day 7. Data presented as mean concentration (solid line) and standard deviation (dashed lines). SBECD, sulfobutylether-β-cyclodextrin; CVVH, continuous venovenous hemofiltration; IV, intravenous.

**Figure 3:**
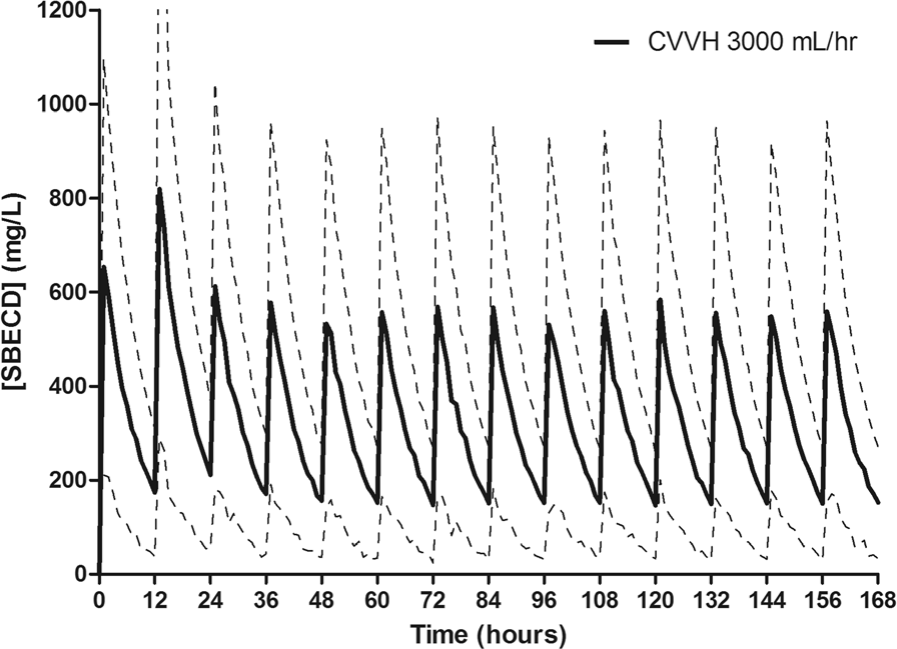
**Simulated plasma SBECD pharmacokinetic profiles in subjects undergoing CVVH with an ultrafiltration rate of 3,000 ml/hr.** Data represent 1,000 patient simulations for SBECD exposure with voriconazole 6 mg/kg IV every 12 hours for two doses followed by 4 mg/kg IV every 12 hours from time 0 to day 7. Data presented as mean concentration (solid line) and standard deviation (dashed lines). SBECD, sulfobutylether-β-cyclodextrin; CVVH, continuous venovenous hemofiltration; IV, intravenous.

**Figure 4:**
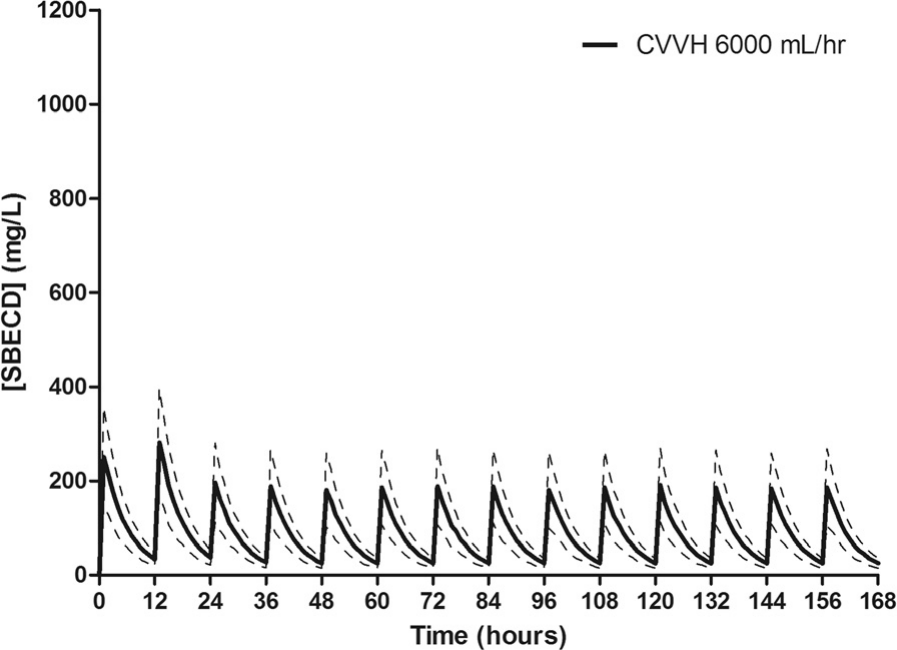
**Simulated plasma SBECD pharmacokinetic profiles in subjects undergoing CVVH with an ultrafiltration rate of 6,000 ml/hr.** Data represent 1,000 patient simulations for SBECD exposure with voriconazole 6 mg/kg IV every 12 hours for two doses followed by 4 mg/kg IV every 12 hours from time 0 to day 7. Data presented as mean concentration (solid line) and standard deviation (dashed lines). SBECD, sulfobutylether-β-cyclodextrin; CVVH, continuous venovenous hemofiltration; IV, intravenous.

A summary of the major SBECD pharmacokinetic variables can be found in Table 3. SBECD was readily removed by CVVH with a median sieving coefficient of 0.85 (IQR 0.52 to 1.1). CVVH accounted for 86% of the total SBECD clearance, and 85% of the SBECD administered was recovered in the effluent fluid. Minimal SBECD plasma accumulation was observed, as demonstrated by nonsignificant changes in dose-normalized SBECD Cmax (703 ± 600 to 684 ± 432 mg/L; *P* = 0.94), Cmin (228 ± 250 to 221 ± 189 mg/L; *P* = 0.94), and AUC0-12 (4,484 ± 4,368 to 4,553 ± 2,880 mg*hr/L; *P* = 0.97) on study day 1 versus days ≥3, respectively. Simulated SBECD plasma concentration versus time curves on days 1 to 7 in all patients and delineated by CVVH ultrafiltration rates of 2,000, 3,000, or 6,000 ml/hr are depicted in Figures 1, 2, 3 and 4 and Figure S1 in Additional file 2. The mean SBECD clearance ranged from 1.75 L/hr at an ultrafiltration rate of 2,000 ml/hr to 4.69 L/hr at an ultrafiltration rate of 6,000 ml/hr.

**Table 3:** **Steady-state plasma pharmacokinetics of SBECD and voriconazole during CVVH**

**SBECD**							
	**Cmax (mg/L)**	**Cmin (mg/L)**	**T1/2 (hrs)**	AUC0-12 (mg*hr/L)	**Cl**_**s**_ **(L/hr/kg)**	**Vd (L/kg)**	**CVVH CL (L/hr/kg)**
Mean	687.0	214.5	6.3	4396	0.03	0.3	0.02
SD	500.4	202.6	1.6	3325	0.02	0.2	0.01
Median	561.1	165.2	6.2	3621	0.02	0.2	0.01
25%	216.2	38.7	5.3	1352	0.01	0.1	0.01
75%	1102.4	305.8	7.3	6804	0.05	0.4	0.02
**Voriconazole**							
	**Cmax (mg/L)**	**Cmin (mg/L)**	**T1/2 (hrs)**	AUC0-12 (mg*hr/L)	**Cl**_**s**_ **(L/hr/kg)**	**Vd (L/kg)**	**CVVH CL (L/hr/kg)**
Mean	4.1	2.4	19.5	37	0.13	3.2	0.02
SD	1.4	1.1	10.7	14	0.06	1.5	0.02
Median	4.1	2.3	19.0	38	0.12	2.7	0.02
25%	3.3	2.0	13.1	30	0.09	2.1	0.01
75%	5.1	3.0	22.5	41	0.14	3.7	0.02

SBECD, sulfobutylether-β-cyclodextrin; CVVH, continuous venovenous hemofiltration; Cmax, maximum drug concentration in the plasma; Cmin, minimum drug concentration in the plasma; T1/2, half-life; AUC0-12 area under the concentration-time curve from time 0 to 12 hours; Cl_s_, total systemic clearance; Vd, volume of distribution; CVVH CL clearance by continuous venovenous hemofiltration; SD, standard deviation.

Voriconazole pharmacokinetic variables are presented in Table 3. The voriconazole half-life (T1/2) was similar amongst intermediate, extensive, and ultrarapid metabolizers (18.3 hrs vs. 19.9 hrs vs. 21.4 hrs, respectively; *P* = 0.86). No patients had the poor metabolizer phenotype. The median fraction of voriconazole clearance attributable to CVVH was 15% (IQR 12 to 21%). The median amount of voriconazole recovered in the effluent fluid was 14% (IQR 11 to 19%) and closely corresponded to the fraction of voriconazole clearance due to CVVH.

The median (IQR) voriconazole N-oxide metabolite Cmax, AUC0-12, voriconazole/voriconazole N-Oxide AUC0-12 and Cmax ratios were 0.9 mg/L (0.4 to 1.5 mg/L), 11.9 mg*hr/L (5.0 to 20.7 mg*hr/L), 2.8 (2 to 4.6), and 3.6 (2.6 to 11.1), respectively. Mean plasma voriconazole N-oxide Cmax, AUC0-12, voriconazole/voriconazole N-oxide AUC0-12 and Cmax ratios were similar among intermediate (1.4 mg/L, 13.9 mg*hr/L, 2.6, and 2.9), extensive (1.3 mg/L, 16.4 mg*hr/L, 6.3, and 8.5), and ultrarapid metabolizers (0.9 mg/L, 10.3 mg*hr/L, 3.0, and 4.0). Voriconazole N-oxide was readily removed by CVVH with a mean ± SD sieving coefficient of 0.8 ± 0.2.

IV voriconazole at standard dosages produced steady-state voriconazole plasma trough values >1 mg/L for all patients. The probability of achieving *f*AUC/MIC >25 with a maintenance dose of 4 mg/kg every 12 hours was >90% for *A. fumigatus* and all *Candida* species except *C. glabrata* (Table 4). Maintenance voriconazole doses of 6 mg/kg every 12 hours were required to achieve >80% target attainment for *A. niger* and *A. terreus*.

**Table 4:** **Probability of pharmacodynamic target attainment**

		***C. albicans***	***C. glabrata***	***C. krusei***	***C. parapsilosis***	***C. tropicalis***	***A. fumigatus***	***A. niger***	***A. terreus***
Pfizer									
4 mg/kg q12h	*f*AUC/MIC >25	99.9	86.3	97.9	99.5	99.6	97.1		
	*f*AUC/MIC >35	99.8	81.6	94.8	99.2	99.3	92.8		
6 mg/kg q12h	*f*AUC/MIC >25	99.9	90.0	99.1	99.7	99.8	99.2		
	*f*AUC/MIC >35	99.8	86.8	98.3	99.6	99.6	97.9		
EUCAST									
4 mg/kg q12h	*f*AUC/MIC >25	99.2					90.3	77.2	60.5
	*f*AUC/MIC >35	99.1					80.3	61.9	41.6
6 mg/kg q12h	*f*AUC/MIC >25	99.4					96.3	91.7	81.4
	*f*AUC/MIC >35	99.2					92.1	80.3	65.4
8 mg/kg q12h	*f*AUC/MIC >25							96.4	89.5
	*f*AUC/MIC >35							90.6	79.0
10 mg/kg q12h	*f*AUC/MIC >25							98.6	94.1
	*f*AUC/MIC >35							95.4	86.1
12 mg/kg q12h	*f*AUC/MIC >25								96.6
	*f*AUC/MIC >35								90.6
CLSI									
4 mg/kg q12h	*f*AUC/MIC >25	99.1	89.1	94.1	99.6	98.0			
	*f*AUC/MIC >35	99.0	86.0	88.1	99.3	97.7			
6 mg/kg q12h	*f*AUC/MIC >25	99.6	92.9	98.0	99.7	98.0			
	*f*AUC/MIC >35	99.5	89.9	95.3	99.6	97.9			

Data reported as % of patients obtaining target *f*AUC/MIC ratio at the prescribed dose. Data not available for every organism within all databases. *f*AUC0-12, free area under the concentration-time curve from 0 to 12 hours; MIC, minimum inhibitory concentration; EUCAST, European Committee on Antimicrobial Susceptibility Testing; CLSI, Clinical and Laboratory Standards Institute.

Patient outcomes were generally consistent with their severity of disease. Six out of ten patients died prior to hospital discharge. Of the four survivors, three patients did not require chronic hemodialysis. No evidence of hepatic or renal disease attributable to SBECD was observed.

## Discussion

Our study demonstrates that SBECD is readily removed by CVVH allowing for the use of IV voriconazole without significant SBECD accumulation. CVVH was responsible for greater than 86% of the total systemic SBECD clearance and this corresponded to a similar rate of SBECD recovery in the effluent fluid. The finding that SBECD is removed by extracorporeal filters is similar to previous studies of intermittent hemodialysis modalities [4,15,17]. An evaluation of four patients receiving IV voriconazole during intermittent hemodialysis demonstrated that SBECD can be effectively removed by dialysis. Unfortunately, SBECD still accumulated between dialysis sessions and SBECD trough concentrations were >400 mcg/ml in three of the four patients by days 10 to 13 of therapy [15]. In a more recent study of 15 patients with end-stage renal disease undergoing a 6-hour treatment with Genius dialysis, standard hemodialysis, or hemodiafiltration, it was found that all three modalities were effective at removing SBECD with approximately two-thirds of the administered dose being recovered in the effluent. However, pharmacokinetic simulations after repeated doses demonstrated that even if intermittent hemodialysis with high-flux filter membranes was conducted every 24 to 48 hours, SBECD exposure would still be 6- to 7-fold higher than in patients with normal renal function [17]. Unlike these intermittent modalities that allow for drug accumulation between dialysis sessions, the use of CVVH in our study prevented further accumulation of SBECD.

Overall, plasma SBECD exposure was higher than in previous studies of healthy volunteers with a mean Cmax of 687 mg/L vs. 458 mg/L and AUC0-12 of 4396 vs. 919 mg*hr/L, respectively [3]. CVVH resulted in a mean SBECD terminal T1/2 of 7 hours, which is prolonged compared to healthy patients with an average glomerular filtration rate of 120 ml/min (healthy subject T1/2 of 1.6 hours) [3]. In our patients undergoing CVVH, steady-state concentrations were reached by approximately 48 hours into therapy. No further drug accumulation was demonstrated after this time period. The volume of distribution was similar to healthy volunteers (0.3 vs. 0.2 L/kg, respectively). The total systemic clearance was reduced: 0.03 L/hr/kg vs. 0.11 L/hr/kg in healthy volunteers [3]. This reduction in clearance is expected since prescribed CVVH ultrafiltration rates (approximately 50 ml/min) were significantly lower than a healthy volunteer kidney with an estimated CrCl of 120 ml/min. In study patients, the SBECD clearance correlated well with the CVVH ultrafiltration rate, and SBECD exposure was similar to values previously observed in patients with moderate renal impairment (CrCl approximately 50 ml/min); where 4-fold increases in AUC and a 50% increase in Cmax is observed when compared to patients without renal dysfunction [3]. Based upon the results of our study, prescribing a predilution CVVH ultrafiltration rate of at least 3,000 ml/hr (approximately 37 ml/kg/hr) will correlate to plasma concentrations and systemic SBECD clearance similar to a patient with an estimated CrCl of 50 ml/min [17]. Ultrafiltration rates of 6,000 ml/hr are required to achieve plasma SBECD exposure similar to patients without renal dysfunction [3].

The finding that SBECD can be effectively removed by CVVH is clinically important, because some cyclodextrins have been associated with hepatotoxicity or nephrotoxicity due to vacuolation [3]. Although our study was small, no evidence to suggest SBECD as a cause of hepatotoxicity or nephrotoxicity was demonstrated in our study patients. This finding is consistent with other SBECD safety studies in humans [3,18]. Additionally, animal studies have only been able to demonstrate cyclodextrin toxicities when dosages more than 50-fold greater (3,000 mg/kg) than those used in humans were administered [3,19,20]. Unlike other cyclodextrins used in these animal studies, SBECD undergoes only minimal tubular reabsorption and limits concentrations within the intracellular tissues of the kidney, potentially reducing the risk of nephrotoxicity. Nevertheless, the FDA labeling for voriconazole recommends that IV therapy be avoided, if possible, in patients with a CrCl <50 ml/min [5]. Our data suggest that IV voriconazole can be safely administered in this population if the patient is concurrently undergoing CVVH.

In our study the mean plasma voriconazole Cmax, Cmin, T1/2, and AUC0-12 were slightly higher than those reported in healthy volunteers receiving 4 mg/kg IV every 12 hours. Similarly, slight alterations in volume of distribution and clearance were also observed [7]. However, it is common for critically ill patients to demonstrate altered pharmacokinetics compared to healthy volunteers, and it is important to note that voriconazole plasma pharmacokinetics during CVVH in our study patients were similar to previous studies of critically ill patients [8,21]. These pharmacokinetic alterations, plus multiorgan dysfunction in many of our patients and the lack of patients with the poor metabolizer phenotype, likely explains why substantial differences in pharmacokinetic parameters between the different CYP2C19 phenotypes were not observed [22,23]. Although considerable variability in voriconazole pharmacokinetics did exist between patients, standard dosages of IV voriconazole were effective at achieving steady-state voriconazole plasma trough levels >1 mg/L in all patients. Pharmacodynamic target attainment was achieved with this dosing strategy for the majority of pathogens within the databases evaluated. These findings suggest that IV voriconazole 6 mg/kg IV every 12 hours for two doses followed by 4 mg/kg IV every 12 hours provides an effective strategy for the treatment of susceptible fungal pathogens in critically ill patients. However, higher doses may be required for less susceptible pathogens including *C. glabrata*, *A. niger*, *and A. terreus*.

Contrary to SBECD, voriconazole elimination by CVVH accounted for only a small and clinically irrelevant percentage (defined as a fraction of clearance due to CVVH of <25%) of the voriconazole total body clearance. This finding is consistent with its predominant extrarenal elimination and is similar to the findings of two recent studies evaluating patients undergoing CRRT while receiving IV voriconazole [8,21]. Therefore, no dosage adjustment is required for voriconazole in patients undergoing CVVH. The voriconazole N-oxide metabolite was readily cleared by CVVH resulting in plasma concentrations and voriconazole/voriconazole N-oxide concentration ratios similar to those previously reported in patients without kidney dysfunction [13,14]. The clinical implications of these findings are yet to be determined as the voriconazole N-oxide metabolite is absent of antifungal activity and has not been linked to adverse clinical effects.

Potential study limitations need to be considered when interpreting our findings. The sample size of our study was small. However, our study currently represents the largest and most comprehensive study of IV voriconazole in patients undergoing CVVH to date. Our study utilized only one mode and type of extracorporeal machine for delivering CRRT. Therefore, the results must be extrapolated to institutions that use different extracorporeal filters, ultrafiltration rates, or post-dilution modes. Nevertheless, it is likely based on previous studies that SBECD will be well removed by most modern high-flux filters and dialysis modes. It is also improbable that voriconazole will be removed by CRRT to an extent requiring dosage adjustment. Due to the severity of illness and high rate of mortality in our study, evaluation of adverse events was limited to only four patients.

## Conclusions

CVVH effectively removed SBECD at a rate similar to the ultrafiltration rate. Voriconazole clearance by CVVH was not clinically significant. Standard recommended dosages of IV voriconazole are effective at achieving goal plasma concentrations for most pathogens and can be utilized in critically ill patients undergoing CVVH without significant risk of SBECD accumulation.

## Key messages

- CVVH effectively removes SBECD and allows for the use of IV voriconazole without the risk of SBECD accumulation
- Voriconazole removal by CVVH was minimal and standard IV dosages were effective at maintaining trough levels >1 mcg/ml in these critically ill patients
- Voriconazole dosages >4 mg/kg every 12 hours may be necessary to obtain pharmacodynamic targets for pathogens with higher MICs, including *A. niger* and *A. terreus*

## Additional files

Additional file 1:
**Detailed study methodology.**
Additional file 2: Figure S1. Simulated plasma SBECD pharmacokinetic profiles for: all subjects, patients undergoing CVVH with an ultrafiltration rate of 2,000 ml/hour, 3,000 ml/hour, and 6,000 ml/hour. Data represent 1,000 patient simulations for SBECD exposure with voriconazole 6 mg/kg IV every 12 hours for two doses followed by 4 mg/kg IV every 12 hours from time 0 to day 7. Data presented as mean concentration (solid lines) and standard deviation (dashed lines).

## Abbreviations

APACHE II: Acute Physiologic and Chronic Health Evaluation II score
AUC0-12: area under the concentration-time curve from 0 to 12 hours
Cl_s_: total systemic clearance
CLSI: Clinical and Laboratory Standards Institute
Cmax: maximum drug concentration in the plasma
Cmin: minimum drug concentration in the plasma
CrCl: creatinine clearance
CRRT: continuous renal replacement therapy
CVVH: continuous venovenous hemofiltration
CYP: cytochrome P450
EUCAST: European Committee on Antimicrobial Susceptibility Testing
*f*AUC0-12: free area under the concentration-time curve from 0 to 12 hours
GI: gastrointestinal
ICU: intensive care unit
IQR: interquartile range
IV: intravenous
LC-MS/MS: liquid chromatography-tandem mass spectrometry
MIC: minimum inhibitory concentration
PTA: probability of target attainment
SBECD: sulfobutylether-β-cyclodextrin
SCr: serum creatinine
SD: standard deviation
SOFA: Sequential Organ Failure Assessment score
T1/2: half-life; ultrafiltration rate or effluent rate; hemofiltration rate + net ultrafiltration
Vd: volume of distribution
