# Supplementary material for: Evaluation of sulfobutylether-β-cyclodextrin (SBECD) accumulation and voriconazole pharmacokinetics in critically ill patients undergoing continuous renal replacement therapy
Source: Crit Care. 2015 Feb 3;19(1):32. doi: 10.1186/s13054-015-0753-8 (PMC4338618; doi:10.1186/s13054-015-0753-8)
Supplement: Additional file 1: — Detailed study methodology. [file 13054_2015_753_MOESM1_ESM.docx]

**Additional File 1: Detailed study methodology**

*Pharmacokinetic analysis*

Plasma and dialysate concentration-time data for SBECD, voriconazole, and voriconazole N-oxide were analyzed by standard noncompartmental pharmacokinetics. Peak drug concentrations in plasma (C_max_) and the times at which these concentrations were achieved (T_max_) were estimated by visual inspection of the plasma concentration-versus-time data. Minimum plasma concentration (C_min_) was determined by direct measurement. The apparent terminal elimination rate constant (k_el_) was determined by least-squares regression analysis of the terminal portion of the natural log concentration-time curve. Elimination half-life (t_1/2_) was calculated as 0.693/k_el_. The area under the concentration-time curve from time zero to the end of the 12-hour dosing interval (AUC_0-12_) was calculated by the linear trapezoidal summation method. Total systemic clearance (CL_s_) was calculated as dose/AUC_0-12_. Since SBECD, voriconazole, and voriconazole N-oxide concentrations were not at steady state during the first sampling period, the volume of distribution (V_d_) was calculated by non-steady state methods; for the second sampling period the steady-state V_d_ was calculated as dose/(k_el_ x AUC_0-12_). Additional pharmacokinetic parameters evaluated were: sieving coefficient (Sc) using the ratio of the ultrafiltrate AUC_0–12_ to the serum AUC_0–12_, clearance by the CVVH (CL_CVVH_) in the predilution mode via the formula CL_CVVH_ = Quf x Sc x [Qb/(Qb+ Qrf)], where Quf is the total ultrafiltration rate (hemofiltration + net ultrafiltration), Qub is the extracorporeal blood flow, and Qrf is the predilution replacement therapy fluid flow rate, and fractional clearance by CVVH (Fr_CVVH_) using the ratio of CL_CVVH_ to total body clearance (CLtot). The amount of voriconazole or SBECD eliminated by CVVH was also estimated from the AUC_0–12_ of the ultrafiltrate concentration–time curve and the ultrafiltration rate. To evaluate degree of drug accumulation dose normalized AUC_0-12_, C_max_, and C_min_ were evaluated on study day 1 versus study days ≥ 3. Simulation of day >3 parameters was conducted in the 2 patients that died prior to completing day 3 pharmacokinetic sampling. Dose normalization (to 64 mg/kg of SBECD) was conducted by proportionally adjusting the AUC_0-12_, C_max_, and C_min_ by the SBECD dose administered in mg/kg. All calculations were made by programming pharmacokinetic equations into Microsoft Excel 2010 (Microsoft Corporation, Redmond, WA) and were validated using WinNonlin version 5.0.1 (Pharsight Corporation, Mountain View, CA). Comparisons of pharmacokinetic parameters on day 1 versus study days ≥ 3 were evaluated with a paired t-test.

*Quantification of SBECD, voriconazole, and voriconazole N-oxide concentrations*

SBECD, Voriconazole, and voriconazole N-oxide concentrations were performed utilizing an LC-MS/MS method developed at the University of Colorado Anschutz Medical Campus Medicinal Chemistry Core Facility.

A validated assay for the measurement of SBECD in plasma was provided by Cydex Pharmaceuticals, Inc. SBECD (Captisol) and the internal standard captisol-G were provided in kind by Cydex Pharmaceuticals, Inc. The lower level of quantification for SBECD was 5 mcg/ml. Liquid chromatography employed a ThermoScientific Hypersil Silica Column 250 x 4.6 mm, 5 micron particle size, column at 40°C with a flow-rate of 0.4 mL/min. The mobile phase consisted of A: 100 mM ammonium acetate in water, and B: 50:50 ACN:MeOH.

Captisol was monitored via electro-spray ionization negative ion mode (ESI -) using the following conditions: i) an ion-spray voltage of -4500 V; ii) temperature, 400 oC; iii) curtain gas (CUR; set at 35) and Collisionally Activated Dissociation (CAD; set at 12) gas were nitrogen; iv) Ion Source gas one (GS1) and two (GS2) were set at 20; v) entrance potential was set at 10.0 V; vi) quadruple one (Q1) and (Q3) were set on Low resolution; vii) dwell time was set at 200 msec; and viii) declustering potential (DP), collision energy (CE), and collision cell exit potential (CXP) are voltages (V). Compound settings were: i) Captisol®, 907.1 → 153.0 m/z, DP = -150, CE = -115, CXP = -1. Liquid chromatography employed a ThermoScientific Hypersil Silica Column 250 x 4.6 mm, 5 micron particle size, column at 40 °C with a flow-rate of 0.4 mL/min. The mobile phase consisted of A: 100 mM ammonium acetate in water, and B: 50:50 ACN:MeOH. Between PK samples, the auto sampler was washed with a 1:1:1:1 mixture of ACN:MeOH:IPA:water containing 0.1% formic acid. The chromatography method used was 95% A for 0.50 min; brought to 95% B at 2.00 min and held for 4.50 min; brought back to 95% A at 7.50 min and held for 2.5 min (10.0 min total run time).

For determining voriconazole and voriconazole N-oxide concentrations, plasma samples were processed as follows: An extraction solution containing the internal standard (IS) ketoconazole was freshly prepared in a 100 mL volumetric flask containing 4:1 (1:1 ACN:MeOH) and water (v/v). In individual sets, the PK sample tubes were removed from the freezer (-80 ± 10 °C) and allowed to thaw on ice (40-45 min). The tubes were vortex mixed (3-5 s) and then a sample (200 μL) was transferred to an eppendorf tube (1.5 mL) and mixed with extraction solution (400 μL), vortex mixed (5 s), sat at RT for 5 min, vortex mixed a 2^nd^ time (5 s), and then centrifuged at 10,000 rpm (10 min) using an Eppendorf minispin centrifuge (Hamburg, Germany). The supernatants were transferred into individual wells of a 96-well plate. The 96-well plate was placed into the LEAP auto-sampler cool-stack (6.0 ± 0.1 ^o^C) and samples (10 μL) were analyzed via LC/MS-MS. An Applied Biosystems Sciex 4000 ® (Applied Biosystems; Foster City, CA) equipped with a Shimadzu HPLC (Shimadzu Scientific Instruments, Inc.; Columbia, MD) and Leap auto-sampler (LEAP Technologies; Carrboro, NC) was used. Liquid chromatography employed an Agilent Technologies, Zorbax extended-C18 50 x 4.6 mm column, 5 micron particle size, equipped with a column guard and operated at 40°C with a flow-rate of 0.4 mL/min. The mobile phase consisted of A: 10 mM ammonium acetate, 0.1% formic acid in water, and B: 50:50 ACN:MeOH.

Voriconazole and voriconazole N-oxide were monitored via electro-spray ionization positive ion mode (ESI+) using the following conditions: i) an ion-spray voltage of 5500 V; ii) temperature, 450 °C; iii) curtain gas (CUR; set at 10) and Collisionally Activated Dissociation (CAD; set at 5) gas were nitrogen; iv) Ion Source gas one (GS1) and two (GS2) were set at 30; v) entrance potential was set at 10.0 V; vi) quadruple one (Q1) and (Q3) were set on Unit resolution; vii) dwell time was set at 200 msec; and viii) declustering potential (DP), collision energy (CE), and collision cell exit potential (CXP) are voltages (V). Compound settings were: i) voriconazole (1): 350.1 → 281.1 m/z, DP = 61, CE = 23, CXP = 18; ii) voriconazole N-oxide 366.1 → 143.2 m/z, DP = 41, CE = 17, CXP = 8; iii) ketoconazole (Internal Standard; IS) 531.1 → 81.2 m/z, DP = 76, CE = 125, CXP = 12. Liquid chromatography employed an Agilent Technologies, Zorbax extended-C18 50 x 4.6 mm column, 5 micron particle size, equipped with a column guard and operated at 40 °C with a flow-rate of 0.4 mL/min. The mobile phase consisted of A: 10 mM ammonium acetate, 0.1% formic acid in water, and B: 50:50 ACN:MeOH. Between samples the auto sampler was washed with a 1:1:1:1 mixture of ACN:MeOH:IPA:water containing 0.1% formic acid. The chromatography method used was 95% A for 0.50 min; brought to 95% B at 6.50 min and held for 2.50 min; brought back to 95% A at 10.50 min and held for 1.5 min (12.0 min total run time). The voriconazole lower limit of quantification was 0.05 mcg/mL and the coefficient of variation was < 7% at all standard curve concentrations.

*Cytochrome P450 (CYP) 2C19 Pharmacogenetic analysi*s

Genomic DNA was isolated from 200 μL whole blood using a QIAamp DNA Mini Kit (Qiagen, Valencia, California). Patients were genotyped in duplicate for *CYP2C19*2* (c.681G>A, rs4244285), *CYP2C19*3* (c.636G>A, rs4986893), and *CYP2C19*17* (c.-806C>T, rs12248560) polymorphisms using PCR-pyrosequencing analysis (PSQ 96 MA, Qiagen, Valencia, California) according to standard manufacturer protocol. The PCR and sequencing primers used were:

- *CYP2C19*2*: PCR-forward, 5’-biotin-TTCCCACTATCATTGATTATTTCC-3’; PCR-reverse, 5’-GTCCATCGATTCTTGGTGTTCT-3’; and Sequencing, 5’-TTAAGTAATTTGTTATGGGT-3’. PCR annealing temperature, 60ºC.
- *CYP2C19*3*: PCR-forward, 5’-AATGAAAACATCAGGATTGTAAGC-3’; PCR-reverse, 5’-biotin-AAAAAACTTGGCCTTACCTGG-3’; and Sequencing, 5’-TGTAAGCACCCCCTG-3’. PCR annealing temperature, 57 ºC.
- *CYP2C19*17*: PCR-forward, 5’-TGGGGCTGTTTTCCTTAGATAAAT-3’; PCR-reverse, 5’-biotin-TGGCGCATTATCTCTTACATCAG-3’; and Sequencing, 5’-TTTGTGTCTTCTGTTCTCA-3’. PCR annealing temperature, 60ºC.

*CYP2C19* genotypes were used to assign CYP2C19 metabolizing enzyme phenotypes according to literature conventions.[PMID 22027650] Briefly, *CYP2C19* *1/*17 or *17/*17 genotypes were classified as ultrarapid metabolizers; *CYP2C19* *1/*1 genotypes were classified as extensive metabolizers; *CYP2C19* *1/*2, *1/*3, or *2/*17 genotypes were classified as intermediate metabolizers; and *CYP2C19* *2/*2, *2/*3, or *3/*3 genotypes were classified as poor metabolizers.

| **MIC** | **0.004** | **0.008** | **0.016** | **0.032** | **0.064** | **0.125** | **0.25** | **0.5** | **1** | **2** | **4** | **8** | **16** | **32** | **64** | **128** | **256** | **512** | **Total Isolates** |
| --- | --- | --- | --- | --- | --- | --- | --- | --- | --- | --- | --- | --- | --- | --- | --- | --- | --- | --- | --- |
| C. albicans Pfizer | 0 | 3314 | 304 | 106 | 22 | 14 | 11 | 13 | 3 | 1 | 1 | 0 | 0 | 0 | 0 | 0 | 0 | 0 | 3789 |
| C. albicans CLSI | 272 | 4048 | 1264 | 511 | 262 | 94 | 50 | 26 | 17 | 10 | 7 | 9 | 9 | 69 | 5 | 8 | 0 | 0 | 6661 |
| C. albicans EUCAST | 14 | 120 | 919 | 176 | 28 | 25 | 24 | 12 | 8 | 4 | 1 | 7 | 8 | 0 | 0 | 0 | 0 | 0 | 1346 |
| C. glabrata CLSI | 5 | 23 | 23 | 72 | 252 | 570 | 626 | 302 | 141 | 101 | 89 | 21 | 2 | 1 | 2 | 0 | 0 | 0 | 2230 |
| C. glabrata Pfizer | 0 | 0 | 3 | 21 | 133 | 251 | 311 | 143 | 50 | 35 | 53 | 18 | 3 | 0 | 0 | 0 | 0 | 0 | 1021 |
| C. krusei CLSI | 0 | 0 | 3 | 12 | 4 | 59 | 235 | 252 | 66 | 14 | 1 | 0 | 0 | 0 | 0 | 0 | 0 | 0 | 646 |
| C. krusei Pfizer | 0 | 1 | 0 | 1 | 7 | 58 | 109 | 54 | 8 | 1 | 1 | 0 | 0 | 0 | 0 | 0 | 0 | 0 | 240 |
| C. parapsilosis CLSI | 0 | 260 | 550 | 335 | 143 | 97 | 64 | 25 | 7 | 2 | 1 | 1 | 0 | 0 | 0 | 0 | 0 | 0 | 1485 |
| C. parapsilosis Pfizer | 0 | 300 | 418 | 136 | 58 | 42 | 28 | 15 | 2 | 4 | 0 | 0 | 0 | 0 | 0 | 0 | 0 | 0 | 1003 |
| C. tropicalis CLSI | 27 | 121 | 249 | 574 | 507 | 170 | 59 | 40 | 10 | 6 | 0 | 4 | 4 | 15 | 3 | 2 | 0 | 2 | 1793 |
| C. tropicalis Pfizer | 0 | 88 | 284 | 271 | 155 | 27 | 11 | 5 | 5 | 0 | 0 | 1 | 0 | 0 | 0 | 0 | 0 | 0 | 847 |
| A. fumigatus Pfizer | 0 | 0 | 0 | 0 | 0 | 13 | 338 | 248 | 33 | 3 | 2 | 0 | 0 | 0 | 0 | 0 | 0 | 0 | 637 |
| A. fumigatus EUCAST | 0 | 0 | 0 | 0 | 5 | 36 | 197 | 759 | 216 | 27 | 15 | 3 | 3 | 0 | 0 | 0 | 0 | 0 | 1261 |
| A. niger EUCAST | 0 | 0 | 0 | 1 | 5 | 2 | 1 | 10 | 18 | 4 | 0 | 0 | 0 | 0 | 0 | 0 | 0 | 0 | 41 |
| A. terreus EUCAST | 0 | 0 | 0 | 0 | 0 | 0 | 10 | 45 | 145 | 62 | 8 | 0 | 0 | 0 | 0 | 0 | 0 | 0 | 270 |

**eTable 1: Isolate numbers and MIC distributions by database utilized for pharmacodynamic modeling**
