# Supplementary figures and images for: Evaluation of sulfobutylether-β-cyclodextrin (SBECD) accumulation and voriconazole pharmacokinetics in critically ill patients undergoing continuous renal replacement therapy
Source: Crit Care. 2015 Feb 3;19(1):32. doi: 10.1186/s13054-015-0753-8 (PMC4338618; doi:10.1186/s13054-015-0753-8)

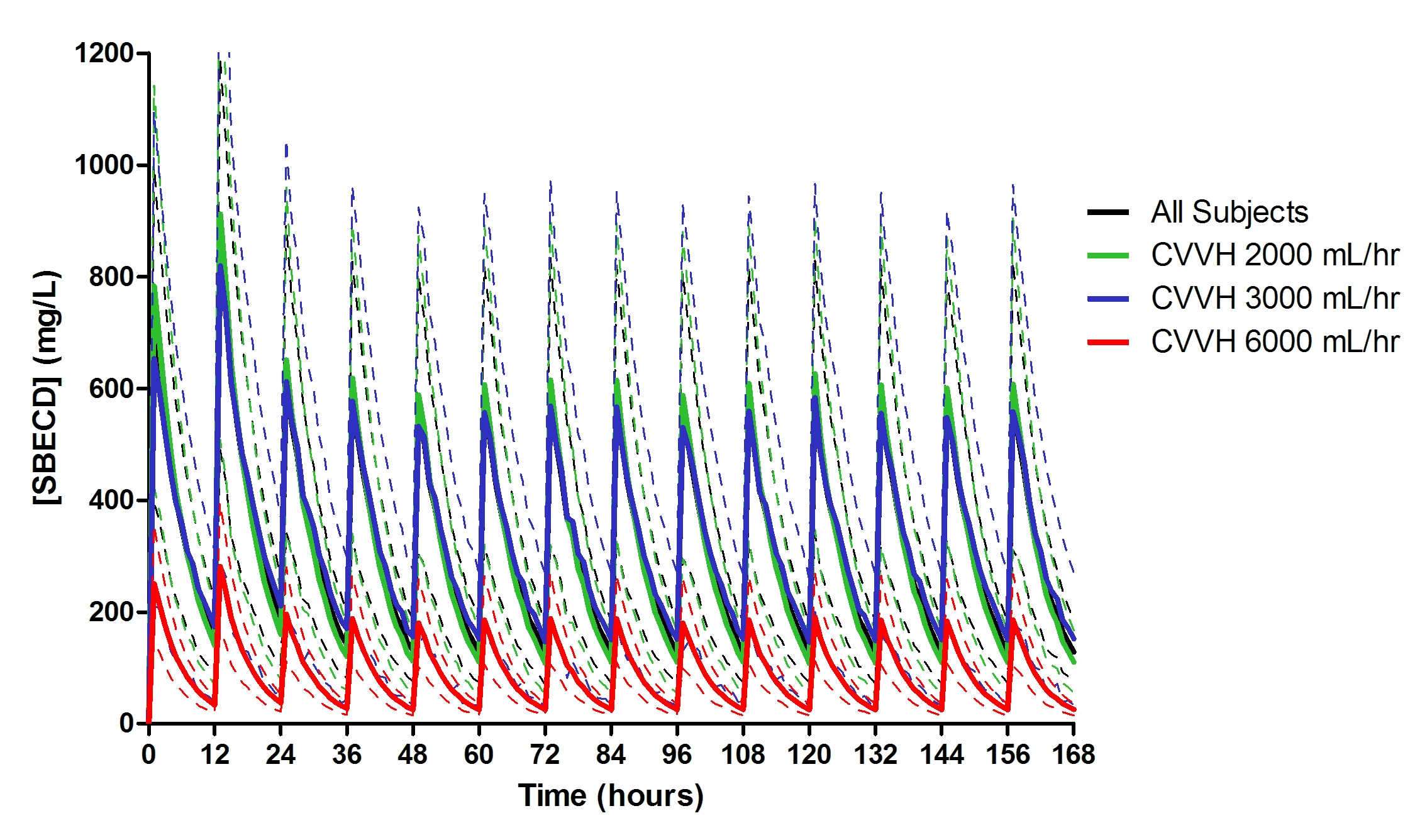

Supplement: Additional file 2: Figure S1. — Simulated plasma SBECD pharmacokinetic profiles for: all subjects, patients undergoing CVVH with an ultrafiltration rate of 2,000 ml/hour, 3,000 ml/hour, and 6,000 ml/hour. Data represent 1,000 patient simulations for SBECD exposure with voriconazole 6 mg/kg IV every 12 hours for two doses followed by 4 mg/kg IV every 12 hours from time 0 to day 7. Data presented as mean concentration (solid lines) and standard deviation (dashed lines). [file 13054_2015_753_MOESM2_ESM.tiff]
